# Microsurgical resection of medullary cavernoma via the olivary zone by the retrosigmoid supracondylar approach

**DOI:** 10.3171/2019.10.FocusVid.19293

**Published:** 2019-10-01

**Authors:** Ken Matsushima, Michihiro Kohno, Helmut Bertalanffy

**Affiliations:** 1 Department of Neurosurgery, Tokyo Medical University, Tokyo, Japan; and; 2 Center for Vascular Neurosurgery, International Neuroscience Institute Hannover, Hannover, Germany

**Keywords:** brainstem cavernous malformation, cerebellopontine angle, far lateral transcondylar approach, posterior fossa, safe entry zone, video

## Abstract

Microsurgical resection of the medullary cavernoma is rare, comprising less than 15% of more than 250 surgeries of brainstem cavernoma performed by the senior author (H.B.).^1^ This video demonstrates a case of a cavernous malformation inside the lateral part of the medulla, which was surgically treated via the olivary zone by the retrosigmoid supracondylar approach in a half-sitting position. Osseous drilling of the lateral foramen magnum provided wide exposure of the cerebellomedullary cistern around the olive.^2,3^ The lesion was completely dissected at the appropriate cleavage plane from the normal parenchyma. The patient developed no new neurological deficits and had no recurrence during 3 years of follow-up after the operation.

The video can be found here: https://youtu.be/7i7SccS5HmU.

## Transcript

This video shows the microsurgical resection of a medullary cavernoma via the olivary zone, by the retrosigmoid supracondylar approach.

### 0:30 Clinical history

A 68-year-old woman first experienced a medullary hemorrhage 5 years before the surgery, and the second hemorrhage occurred 5 months before the surgery. The repeated hemorrhage worsened her neurological symptoms, and she had right facial hypesthesia, dysphagia, dysarthria, slight right hemiparesis, dissociated hemisensory deficit on the right side of her body, and significant gait ataxia.

### 0:57 Preoperative radiological images

MRI showed a 16-mm round lesion in the lateral part of the left medulla, which indicated hemorrhagic cavernoma. The size of the lesion was bigger than that after the first hemorrhage.

### 1:11 Setup and exposure

The retrosigmoid supracondylar approach and access through the olivary zone were planned with the aim of complete lesion removal under MEP, SEP, and hypoglossal electromyography.

In a half-sitting position with slight head flexion, a C-shaped retroauricular incision was made on the left side. A retrosigmoid craniotomy was performed and the lateral rim of the foramen magnum, including the condylar fossa, was drilled out for wide exposure of the cerebellomedullary cistern around the olive. The dura was incised along the sigmoid sinus and the incision was extended inferiorly through the foramen magnum. After releasing the cerebrospinal fluid from the cerebellomedullary cistern, the lower cranial nerves, choroid plexus, PICA, and vertebral artery were carefully dissected. The lateral part of the medulla was carefully observed, while respecting the lower cranial nerves, PICA’s perforators to the medulla, and hypoglossal nerve. You can see the hemosiderin discoloration and slight surface bulging caused by the lesion covered by the healthy parenchyma.

### 2:25 Lesion removal

A pial incision was made into the olive with a puncture just posterior to the origin of the hypoglossal nerve with the medulla on the preolivary sulcus. The lesion was not exophytic but was located right underneath the incision. After identifying an appropriate cleavage plane, the entire surface of the cavernoma was dissected following this plane using fine-tipped bipolar forceps and microscissors. A hook dissector was also useful for bringing the deeper portion of the lesion from the hidden space to the exposed space, and dissection of this large lesion through the tiny cavity. The wall of the cavernoma was removed together, while coagulation was performed to shrink the lesion and to make the wall strong enough to dissect. The surgical field was irrigated with nimodipine solution to prevent local vasospasm. Multiple small arterial feeder vessels into the cavernoma were dissected, coagulated, and transected meticulously one by one. As usual, the most adherent portion was precisely managed at the final stage of the lesion dissection. The lesion was resected completely. You can see the size of the cavity with a millimeter scale and all the surrounding structures, including the cranial nerves and perforators, were completely preserved without any damage.

### 4:14 Postoperative course

Extubation was performed immediately after the surgery and the patient developed no new neurological deficits. On postoperative MRI, you can confirm complete resection of the lesion and reduction of the mass effect. During the 3 years of follow-up after the operation, the patient had no new neurological deficits or recurrence. The modified Rankin Scale improved from 3 before the surgery to 0 on the final follow-up day.

## Disclosures

The authors report no conflict of interest concerning the materials or methods used in this study or the findings specified in this publication.
